# Conceptualization, Development and Psychometric Evaluations of a New Medication-Related Health Literacy Instrument: The Chinese Medication Literacy Measurement

**DOI:** 10.3390/ijerph17196951

**Published:** 2020-09-23

**Authors:** Hsiang-Wen Lin, Elizabeth H. Chang, Yu Ko, Chun-Yu Wang, Yu-Shan Wang, Okti Ratna Mafruhah, Shang-Hua Wu, Yu-Chieh Chen, Yen-Ming Huang

**Affiliations:** 1 School of Pharmacy and Graduate Institute, College of Pharmacy, China Medical University, Taichung City 404333, Taiwan; u102003556@cmu.edu.tw (Y.-S.W.); oktiratnamafruhah@gmail.com (O.R.M.); pharmchen@gmail.com (Y.-C.C.); 2 Department of Pharmacy, China Medical University Hospital, Taichung City 404332, Taiwan; 3 Department of Pharmacy System, Outcomes and Policy, College of Pharmacy, University of Illinois at Chicago, Chicago, IL 60612, USA; 4 Department of Clinical Pharmacy, School of Pharmacy, College of Pharmacy, Taipei Medical University, Taipei City 110301, Taiwan; elizabeth@tmu.edu.tw (E.H.C.); nancyko@tmu.edu.tw (Y.K.); 5 Research Center for Pharmacoeconomics, College of Pharmacy, Taipei Medical University, Taipei City 110301, Taiwan; 6 Department of Pharmacy, Wan Fang Hospital, Taipei Medical University, Taipei City 116081, Taiwan; 7 Department of Pharmacy, Shin Kong Wu Ho-Su Memorial Hospital, Taipei City 111045, Taiwan; Skhpharmacy@gmail.com; 8 Taiwan International Pharmacy Advancement Association, Taipei City 100006, Taiwan; 9 Department of Pharmacy, Songde Branch, Taipei City Hospital, Taipei City 110209, Taiwan; sunnie0225@gmail.com; 10 Graduate Institute of Clinical Pharmacy, College of Medicine, National Taiwan University, Taipei City 100025, Taiwan; 11 Department of Allied and Population Health, College of Pharmacy and Allied Health Professions, South Dakota State University, Brookings, SD 57007, USA

**Keywords:** health literacy, health professionals, medication literacy, psychometric properties

## Abstract

There is a need for valid and reliable instruments to focus on medication aspects of health literacy and help healthcare professionals address patients’ barriers to medication use. This cross-sectional study describes the conceptualization, development, and psychometric properties of the first Chinese Medication Literacy Measurement (ChMLM) to assess the level of health literacy on medication use. The 17-item ChMLM (ChMLM-17) and its short form, 13-item ChMLM (ChMLM-13), consist of four sections (vocabulary, over-the-counter labels, prescription labels, and advertisements) to cover six domains of medication-related health literacy. Multistage stratified quota sampling was attempted to recruit a representative sample in Taiwan. Receiver operating characteristic curves were used to identify the cut-off point for differentiating high and low medication literacy. Psychometric analyses were performed (*n* = 1410) to assess the reliability and validity separately on all samples and sociodemographic subgroups. The 17- and 13-item versions both had high construct validity among all patients and patients with low medication literacy. The developed ChMLM-17 and ChMLM-13 is expected to help healthcare providers and researchers to accurately measure medication-related health literacy and improve medication use in the real-world practice.

## 1. Introduction

Medication use, including the consumption of prescription and over-the-counter (OTC) medications, is a common routine for disease management of patients across ages and countries [1]. For example, around 70% of adults in North America take at least one prescription medication daily [2], and 40–95% of adults used OTCs in the Arab world, Germany, and Africa [3,4,5,6]. Inappropriate use of OTC medications, even if these products are claimed to be safe for self-medication, might cause serious consequences to patients’ health [7,8]. However, studies have shown that patients are often confused about medication information provided by pharmaceutical companies and healthcare professionals [8], and that misinterpretation of medication information (e.g., warning label, package label and insert, and medication instruction pamphlet) is linked to medication nonadherence and misuse [9,10]. Thus, misunderstanding of the dosing instruction on medication packages has been cited as a critical factor that contributes to unintentional medication errors and adverse events [11].

Evaluation of medication information provided by a pharmaceutical company is a way to understand patient ability of critical thinking about medication use [12]. Nowadays, medication information is often communicated through various forms of mass media, and has imposed considerable impacts on patients’ decision-making regarding disease management [13]. Some information delivered in commercials or advertisements may be incorrectly or inappropriately interpreted due to consumers’ misunderstanding of medical terminologies therein [14,15]. Ideally, an individual’s skills to properly evaluate and apply information from advertisements on medications would facilitate appropriate healthcare decision-making.

Earlier studies have shown that people with lower medication-related health literacy tend to use medications incorrectly or inappropriately in terms of the dose, indication, route, or duration [16,17,18]. Consequently, evaluation of one’s medication-related health literacy could be a proxy for medication safety by knowing the degree to which an individual understands the information related to medication use [19]. The term of medication literacy has since been formally defined as “the degree to which individuals can obtain, comprehend, communicate, calculate, and process patient-specific information about their medications to make informed medication and health decisions to safely and effectively use their medications, regardless of the mode by which the content is delivered [20]”. In other words, this definition infers that medication literacy involves a wide range of skills to process medication-related information, such as numeracy, information-seeking, decision-making, evaluation, and application. It also supports the findings from prior studies that the concept of health literacy in a pharmacy setting should be patient-specific and focus on the individual’s capability to use medications correctly and safely [21]. Accordingly, Pouliot et al. suggest that medication literacy is a two-way dialogue between patients and healthcare professionals about the pros and cons of medication use, not merely from the perspectives of healthcare professionals [20]. As such, accurate identification of patients’ levels of mediation-related health literacy may help healthcare professionals tailor interventions to cope with the problems of medication use specific to patients’ needs [22,23].

According to the above definition of health literacy, recent reviews show that the association between health literacy and medication adherence is either weak [24,25,26,27] or inconclusive [28,29] across different illnesses. One reason for this poor association might be because existing instruments of health literacy are too general to capture wide ranges of abilities specific to medication use [20]. Although some health literacy instruments (e.g., either general health literacy [12], disease-specific [30,31,32,33,34,35], or having certain related domains [36,37,38]) are available, none of these instruments/measures or domains are specifically designed to assess the levels of medication-related health literacy. A few instruments have been developed to assess levels of medication-related health literacy, including the 14-item Medication Literacy Assessment (MedLitRxSE) [39], 20-item Numeracy Understanding in Medicine Instrument (NUMi) [40], 21-item Montana State University Complementary and Alternative Medicine (MSU CAM) Health Literacy [41], and 6-item Medication Health Literacy Measure (MHLM) [42]; the application of these instruments is restricted due to their limited domains covered. Moreover, some contents of these instruments might be distant from day-to-day use in patient care or in community settings. In other words, it would be beneficial to have a medication-specific health literacy instrument to deal with the complex tasks related to medication therapy in clinical practice [20,43].

Subsequently, we developed the first Chinese Medication Literacy Measurement (ChMLM) to capture more aspects of patients’ health literacy relevant to medication use in future clinical practice. Preliminary validation data, which focused on the responses from the convenience sample of 602 recruited participants, provided support for continuing the planed large-scale, nationally-representative validation study with the full instrument and developing a short-form ChMLM [44]. This study aimed to describe the comprehensive development process of this instrument, including the conceptual framework, process of item development and reduction, scoring and application, and to share the instrument, its short form, as well as the English version for further use in real world practice or research.

## 2. Materials and Methods

### 2.1. Conceptual Framework for Item Development of the ChMLM

**Figure 1:**
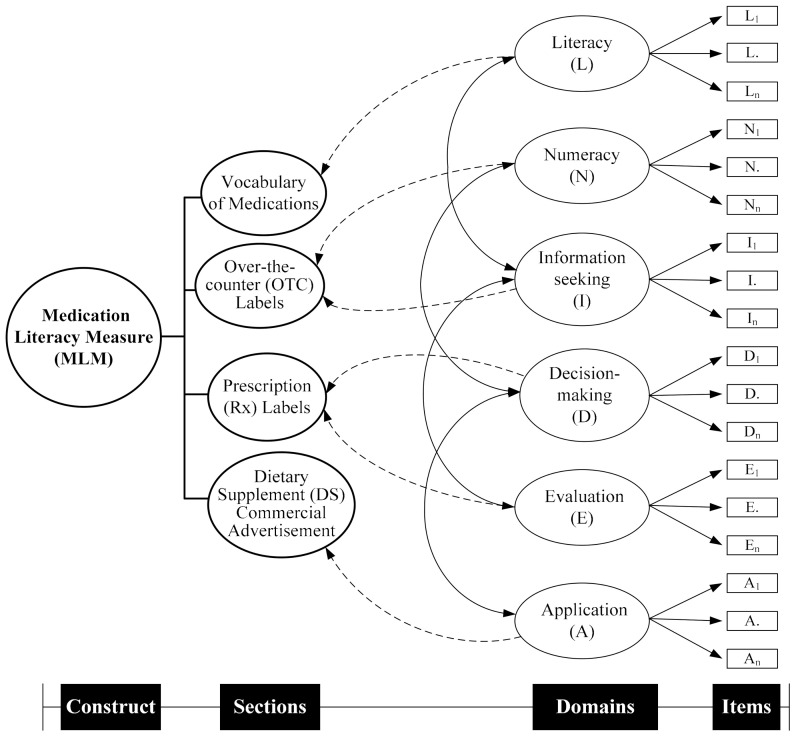
Conceptualization of Medication Literacy Measurement. Note: L_1_, L., L_n_ were items developed to assess literacy; N_1_, N., N_n_ were items developed to assess numeracy; I_1_, I., I_n_ were items developed to assess information-seeking; D_1_, D., D_n_ were items developed to assess decision-making; E_1_, E., E_n_ were items developed to assess evaluation; A_1_, A., A_n_ were items developed to assess application.

**Figure 2:**
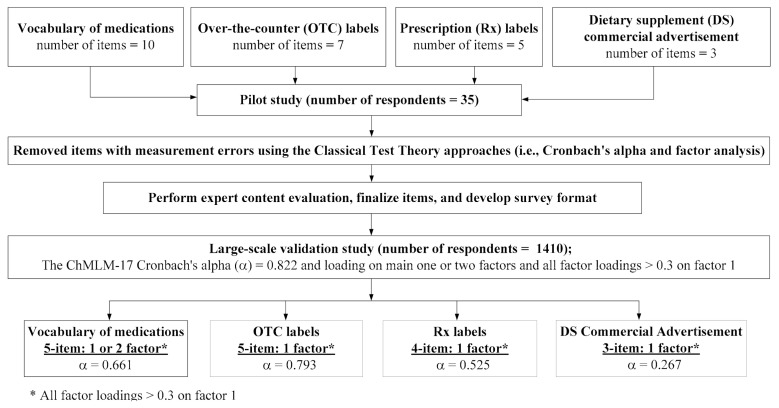
Item reduction process and its psychometric properties of the ChMLM-17.

Based on the study of Sauceda et al. on the conceptualization of medication literacy [39], we adopted their definition of medication literacy as “the ability of individuals to safely and appropriately acquire, understand, and act upon basic medication information [39]” during the development of this instrument in 2015, as the report by Pouliot et al. was not published at that time [23]. Medication information often appears on patients’ education sheets, medication instruction labels, and advertisements in Taiwan. Therefore, it is imperative to expect that patients could read, navigate, understand, and evaluate the information on these aforementioned resources. Accordingly, the ChMLM, as the construct of interest, was proposed to encompass the following four sections related to medication information presented in medication labels, package inserts, or statements on pharmaceutical products and dietary supplements (Figure 1): vocabulary of medications, OTC labels, prescription (Rx) labels, and dietary supplement (DS) commercial advertisements. While the developed items were assumed to assess the common six literacy-related domains (i.e., literacy, numeracy, information-seeking, decision-making, evaluation, application), ten, seven, five, and three items were initially generated for the aforementioned four sections, respectively (Figure 2) by adapting previously validated medication-related literacy measures obtained from a literature review by one of the authors (HWL) [13,39,45,46,47,48,49].

After pilot testing on 35 participants of the convenient samples, the findings of psychometric analyses were combined with the expert content evaluation to finalize the original measure (Table 1) [44], and to develop an appropriate format of the 17-item Chinese Medication Literacy Measurement (ChMLM-17) layout (e.g., please see Supplementary File S1 for images of an OTC package label and Rx labels on the Rx bag and box). Table 1 describes the original item stems. The items in the section on vocabulary mainly contained the literacy domain, and the sections on OTC drug, Rx labels, and DS commercial advertisements comprised mainly the information-seeking domain (Table 1). A few items were more complex and underwent evaluations encompassing more than two subdomains (e.g., one item in the OTC label and two items in the dietary supplement commercial advertisement sections for the I, L, E subdomains, respectively). The item content to be assessed included common ideas about medication use from medication labels or package inserts for the OTC drug, Rx drug, or DS commercial advertisement sections.

### 2.2. Assessments of Item Performance

#### 2.2.1. Sample and Data Collection

A total of 1410 participants in a nationwide face-to-face survey conducted under the research project “Patient Medication Safety Knowledge Network Subproject—Medication Literacy”, from September 2015 to December 2016, served as the samples of this investigation. The national survey participants were 20 years of age or older, able to read and communicate in Mandarin or Taiwanese, and able to provide an informed consent. Those who had problems speaking or hearing or who had a cognitive impairment assessed by the interviewers were excluded from this survey. Participants were recruited from nine randomly selected metropolitan areas, which represented four geographic regions in Taiwan: the north region included Taipei (Taipei City, New Taipei City, Taoyuan, and Chinju); the east region included Hualien; the central region included Taichung City; the south region included Chiayi, Tainan, and Kaohsiung/Pingtung. A multistage stratified quota sampling was used to ensure that participants from each region had similar backgrounds in terms of age, gender, and, if possible, characteristics similar to the population they represented. Potential participants living in the nine cities were referred to surveyors by local leaders or persons-in-charge in community centers, owners of street shops, schoolteachers, pharmacists in community pharmacies, or participants themselves. Other than the student interviewers’ friends, relatives, neighbors, and the customers/members of participating pharmacies and organizations were approached as potential participants [44]. The remaining potential participants were contacted via either postcards or referred by referees to determine their willingness to be interviewed in either their homes or in a safe, undisrupted public or private location.

#### 2.2.2. Instrument

The instructions and layout of the ChMLM-17 were prepared based on the findings and consensus of the research team (presented in Supplementary File S1). The corresponding English version was revised and adapted based on the ChMLM-17 (Supplementary File S2). Specifically, the verbiage and corresponding figures were revised to be more generally applicable to different countries. The statements and contents were rephrased by two native English speakers. Instructions on how to use the instrument and its application (including correct answers and scoring) were prepared as well to facilitate appropriate use under different circumstances or in different countries (Supplementary File S3).

#### 2.2.3. Analyses of Item Performance

All responses were collected and analyzed after completion of the study. The psychometric properties, including reliability, item-total correlation coefficients, and construct validity using exploratory factor analysis, of the whole four-section ChMLM-17 were assessed. The receiver operating characteristic curve (ROC) is a plot of the true positive rate versus the false positive rate for all possible cut-off points [50], and the ROC analysis was performed to decide the cut-off point to categorize high or low medication literacy based upon the education status of college education or above versus high school education or below. Accordingly, the area under the curve (AUC) and its 95% confidence interval (CI) for the ROC were recorded, where an AUC value ≥ 0.7 was set up as an indicator of acceptable discrimination ability [50,51]. Furthermore, the highest sum of sensitivity and specificity was used to decide a cut-off point that could categorize a high or low medication literacy level by maximizing the overall correct differentiation and minimizing the overall misclassification [52].

While the items for the original section of Rx labels were generated based upon the Taiwan-specific pharmacy practice scenario (Supplementary File S1), the content was revised to be commensurate with practice environments in countries that adopt the English Medication Literacy Measurement (MLM; Supplementary File S2). The sequence of Rx labels and DS commercial advertisements in the original ChMLM was reversed to make the instrument easier to use. Importantly, it is recognized that the section of Rx labels might not fit well with the medical care practice in many other countries. Thus, this Rx section should be assessed separately or excluded from the instrument when applicable. Accordingly, the corresponding short-form of the ChMLM, namely ChMLM-13, containing 13 items in three sections (i.e., vocabulary of medications, OTC labels, and DS commercial advertisements) was proposed, and its psychometric properties were evaluated and recorded based on responses from the Taiwanese participants, which were also compared with that of the four-section ChMLM-17 instrument.

#### 2.2.4. Evaluation of Psychometric Properties

The total score of the ChMLM was calculated based on the sum of the number of questions answered correctly (a score of 1 was assigned for each correct answer). Internal consistency was tested by Cronbach’s alpha, in which a coefficient value equal to or greater than 0.70 is considered acceptable [53]. Independent *t*-tests were performed to test the hypotheses that the mean total score of the ChMLM-17 for all participants did not differ across participants with different demographics, self-reported health care utilization, and self-reported health literacy. These hypotheses tests were used to assess discriminant and convergent validity. Specifically, these self-reported questions, including the four-item self-reporting health care utilization (e.g., medical care utilization in the past 3 months and currently taking medications) and the 8-item self-reported health literacy (e.g., knowing the name(s) and health effect(s) of each of the taken medications, and ability to understand information provided by healthcare professionals) were prepared for validation purpose, as alluded in our previous publication [44]. Thus, all responses toward these questions were categorized as dichotomous responses, e.g., <50 year old or ≥50 year old for the age group, with or without doctor visits for medical care utilization in the past three months; <50% or ≥50% perception of knowing the name(s) of used medications; <50% or ≥50% perception of knowing the name(s) of used medications, to compare and contrast their levels of medication literacy, accordingly. Further analyses to stratify the high and low medication literacy groups were performed to examine the discriminant and convergent validity in these two groups. All analyses were conducted using IBM SPSS Statistics 25 (IBM Corp., Armonk, N.Y., USA), with the statistical significance level set at a two-sided *p* < 0.05.

### 2.3. Ethical Considerations

This study was approved by the Institutional Review Board of the China Medical University Hospital, Taiwan [CMUH104-REC3-013]. A signed informed consent was required for each participant before taking part in this study.

## 3. Results

### 3.1. Participant Characteristics

Of 1410 participants who provided complete responses in the national survey, the majority (*n* = 827, 58.65%) were female with age ranging from 20 to 90 years old (mean age = 45.18 ± 17.05) (Supplementary File S4). More than half of the participants (*n* = 723, 51.28%) reported having received a college education or above. Participants were recruited equally from the northern, southern, central, and western regions of Taiwan. Around 65% of the participants followed a religion, and most of them (*n* = 1292, 91.63%) used Mandarin as their major language for communication. More than half of the participants (*n* = 865, 61.35%) reported having an annual individual income of less than USD 10,000.

The majority of the participants had visited a doctor at least once over the past three months (*n* = 965, 68.44%), were taking medications during the study period (*n* = 860, 60.99%), did not need help in taking medications (*n* = 1219, 86.45%), and had experience in taking complementary medications (*n* = 1177, 83.47%) (Supplementary File S5). Although one-third of the participants did not know the names of medications they were taking, more than 80% of the participants understood the effects of the medications they were taking (Supplementary File S6). The majority of the participants had no difficulty in understanding the information provided by healthcare professionals, and had asked healthcare professionals medication-related questions. Most of the participants reported no difficulty in taking medications or understanding the printed information related to medication use. Less than 10% of the participants indicated that they had no confidence in filling out medical forms or understanding health-related information from medical professionals.

### 3.2. Item Performance

Given that the original concept was to divide the measurements into four sections, the distinct Cronbach’s alpha values for the corresponding reliability and its exploratory factor analysis in each section showed that the sections of vocabulary of medications and OTC drug labeling might be more reliable and valid than the other two sections. However, our analysis indicated that these two sections alone were still not sufficiently reliable and valid (Figure 2). Thus, we decided to consider the whole 17 items as only one factor. While the first part of the validation finding published in 2017 showed sufficient psychometric properties based on the first 634 enrolled participants (e.g., Cronbach’s alpha = 0.72, sufficient construct validity) [44], the extent of reliability increased to 0.822, and loadings on the same factor were almost all more than 0.3, based on the responses obtained from all participants in this study (Table 2). Importantly, the values of Cronbach’s alpha for the sections on vocabulary, OTC, and Rx labels increased considerably, which, however, was not noted in the section on DS commercial advertisements, from the small scale pilot study to the large-scale validation study. Obviously, Q17, which was the item addressing the side effects of DS, contributed to the measurement error but not significantly (increasing Cronbach’s alpha to 0.831). Nevertheless, all these lines of evidence support that the whole ChMLM-17 should be recognized as only one factor/domain.

While the mean score of the ChMLM-17 was 11.98 (SD = 3.68), the maximum summary of sensitivity and specificity assessed for the total ChMLM-17 scores were between 12.5 and 13.5 (Table 3). The corresponding AUC value was 0.723 (95% CI: 0.697–0.750), which was greater than 0.7. In this case, 13 was chosen as the cut-off point for the ChMLM-17. When the Rx section was removed from this measure, i.e., the short form of ChMLM-13 with a total score of 13, the reliability reduced to 0.787. Again, Q17 was still the item that contributed most to the measurement error in the ChMLM-13. The ROC of the ChMLM-13 for the cut-off point analysis showed that 10 was the best cut-off-point, with an AUC value of 0.714 (95% CI: 0.687–0.741) for the ChMLM-13.

In terms of individual item performance, there were two main factors, but all the items had strong enough loading on the same factor (Table 3), except for Q17, which belonged to the third factor. The item-correlation coefficients (point-biserial correlation coefficient) between the binary response of each item of the ChMLM-17 and total score were all more than 0.3 (moderate), except for Q17. The correction rate of Q17 was the lowest (27.1%), whereas the mean correction rate was 70.47 ± 16.76% (median 75.9% and interquartile range 63.4 to 83.0%). In this case, it is necessary to reconsider whether to keep Q17 in the ChMLM-17 or to revise Q17 to make the construct more reliable and valid. After further expert content evaluations, we still confirmed that Q17 was very important to keep, but needed rephrasing to make it clear for better readability.

Furthermore, the comparisons of the ChMLM-17 total score with participants’ various characteristics showed that participants with the following characteristics significantly tended to have higher total ChMLM-17 scores (Table 4): namely, more educated; less than 50 years of age; living in the northern region; no religious beliefs, mainly Mandarin speaking; students; currently not taking medications; using complementary and alternative medicine; perceived knowing more than 50% names of drugs; perceived knowing more than 50% of effects of taken medications; no difficulty understanding information provided by healthcare professionals; no difficulty asking health professionals medication-related questions; no difficulty taking medications; perceived understanding of more than 50% of Rx labels, medication package/box labels, and package insert labels; felt confident filling out medication forms; understanding written instructions provided by hospital.

Of all participants, more than half participants (57.10%) were grouped into the high medication literacy group (*n* = 805) and 42.91% (*n* = 605) in the low medication literacy group, based upon their total scores of the ChMLM-17. Further stratification analyses also revealed that the discriminant and convergent validity patterns were similar among those in the low literacy group and all samples (all *p*-values < 0.05). However, these patterns were not observed in the high literacy group for the majority of the demographic variables, with regard to self-reported health literacy and some variables on self-reported health care utilization. All these aforementioned data also confirmed the discriminant and convergent validity of the ChMLM-17.

## 4. Discussion

This study described the comprehensive development process of the first Chinese medication literacy instrument specifically designed to assess patients’ or general public’s levels of medication-related health literacy on medication labels or information of OTC, dietary supplement, and prescription. Our analyses demonstrate that the four-section ChMLM-17 has a good internal consistency, content validity, and construct validity, as does the three-section ChMLM-13.

The diversity of medication-related health literacy instruments has given rise to inconsistencies in measurement and has led to complexities in interpreting findings across different studies and choosing appropriate tools for new research [12]. Variations among different tools may reflect their emphases on distinct conceptual dimensions of medication-related health literacy or medication literacy. To date, no single instrument has been developed to cover the full range of the aforementioned dimensions of medication-related health literacy. Each measurement assesses certain dimensions of medication-related health literacy. Hence, it is important to determine an appropriate instrument that measures the different aspects of medication-related health literacy and reflects each of the skills related to medication use. The existing instruments of medication-related health literacy (i.e., the MedLitRxSE, NUMi, and MSU CAM Health Literacy) use at least 20 items to measure selective domains of medication-related health literacy, focusing mainly on document literacy and numeracy skills rather than addressing other relevant skills needed for medication use, such as information-seeking and decision-making [39,40,41]. While the 6-item MHLM identifies prose literacy, numeracy, and documentation literacy on prescriptions only [42], we developed this ChMLM-17 that encompasses fewer items but captures more domains related to medication literacy (i.e., literacy, numeracy, information-seeking, decision-making, evaluation, and application) than the other aforementioned medication-related health literacy measurements. Indeed, medication use involves a complex set of skills beyond basic comprehension and numerical operation. Using an instrument that measures a wider range of medication-related health literacy could provide healthcare professionals with a deeper understanding of patient skills regarding medication use, and could facilitate tailored interventions to address patients’ barriers to appropriate medication use in daily practice in health or community settings.

The developed ChMLM-17 used vivid scenarios (easier for healthcare professionals to identify what to address/improve) that patients commonly encounter in medication uses, and can serve as a screening tool to identify which aspects of medication use could be potential problems for individuals with low medication literacy. For example, healthcare professionals may use a plain language to highlight the information on the package labels with colors or pictures if patients do not know how to interpret the information on medication bottles [54]. Additionally, healthcare professionals could refine medication information by using visual cues, bullet points, and chunking information to facilitate the readability and understanding of information for medication use [9,55,56,57]. In the future, the developed ChMLM-17, ChMLM-13, and the English version of the MLM could be used to provide a proxy to help healthcare professionals identify patient-related barriers to medication use and facilitate effective counseling or interventions to improve better medication use and ultimately prevent unintentional harm of inappropriate/incorrect medication use due to the misinterpretation of medication information.

### 4.1. Strength and Limitations

This study described the conceptualization, development, and psychometric evaluations of a new medication-related health literacy instrument to assess patients’ or the general public’s levels of medication-related health literacy. However, there were some limitations. For instance, the Q17, exploring the implications of dietary supplement-related side effects in commercial advertisements, was difficult for respondents and may not generate reliable information for analyses. However, after further content mapping, we decided to keep Q17 for content importance, but it will require further revision and testing in future studies. Based upon all these findings, the English version was proposed. The English version of the MLM has not yet been validated, and there may be local differences in the response to medication use, as the interpretation of medication information is related to the social context and availability of information and resources, especially prescription drug labels. The wording, contents, and corresponding images of labels in the English version were also revised and rephrased to allow for more general use in different countries, if applicable (Supplementary File S2). Furthermore, the scoring instructions of the MLM are provided in the Supplementary File S3 to facilitate appropriate use in other contexts or countries. Lastly, while age, gender, and region were comparable to the population in Taiwan in this study, education and other characteristics were not. Thus, the generalizability of these study findings is limited as the study subjects were well-educated and had high general literacy levels. It may impact the relevance of the validity and reliability findings among individuals with both limited levels of general and health literacy. The English version of the MLM may need to be further revised for English-language speaker respondents with limited general literacy in the future. For instance, a low-literacy medication education tool developed by Cordasco et al. [58] and the guidance for providers on medication management for individuals with limited health literacy by Agency for Healthcare Research and Quality [59] could be alternative choices to address medication use of people with limited general and health literacy for other studies.

### 4.2. Future Applications

Future research is warranted to focus on more diverse populations (e.g., people with lower education status), and for different disciplines of health professionals to determine if findings from the present study remain applicable. Future studies are also recommended to explore the validity and usability of the English version of the MLM, as well as to develop different language versions and to perform validation studies in different countries. In clinical practice settings, healthcare professionals can use the reliable and valid ChMLM-17, ChMLM-13 or its English version to understand patients’ medication-related health literacy and to identify potential difficulties that patients may encounter with medication use in the future. Afterward, a focus on difficulties in medication use identified by the ChMLM-17 or ChMLM-13 is recommended for the prevention of incorrect medication use.

## 5. Conclusions

This study demonstrates that the developed ChMLM-17 is a valid and reliable instrument specific to assess health literacy relevant to medication use. Both the ChMLM-17 and ChMLM-13 are performance-based instruments (using four or three sections, respectively) that cover six domains of medication literacy (i.e., literacy, numeracy, information-seeking, decision-making, evaluation, and application) for patients and the general public. The English version of the MLM may be used directly in other countries by different health professionals with a similar medication practice environment, especially for those patients with low medication literacy levels. An understanding of the medication-related health literacy of patients or the general public by using the ChMLM-17, ChMLM-13, or its English version is the first logic step to improve correct medication use in real world practice in the future.

## Availability of Data and Materials

The study materials and the detail of all analyses are available from the corresponding author upon reasonable request.

## Figures and Tables

**Table 1:** Item content and original item stem included in the ChMLM-17.

Section	Subdomain	Item Content to be Assessed	Item ID
**Vocabulary of medication**	L	External use	Q1
		External use: should not be taken by mouth	
	L, E	Fixed-dose combination drug	Q2
		Fixed-dose combination drug: duplicate prescriptions	
	L	Dose	Q3
		Dose: the quantity of medication that is taken at one time (e.g., take 500 mg at a time)	
	L	Side effect	Q4
		Side-effect: extra benefits of the treatment	
	L	Active Ingredient	Q5
		Active ingredient: The main component of the medication that provides a therapeutic effect	
**Over-the-counter labels**	I	Indication of OTC drug	Q6
		What is this medication used for	
	I, N, L	Direction to use	Q7
		How should Mr. Chang take this medication?	
	I, L	Number of total tablets contained	Q8
		How many tablets are in this package according to the package label?	
	I, N, L	Expiration date	Q9
		What is the expiration date of this medication?	
	I, L, E	Warnings	Q10
		Based on the warnings on the package insert label, which situation listed below should Mr. Chang pay more attention to when using this medication?	
**Prescription labels**	I, A	Time to take the next drug	Q11
		Mr. Lee took his first tablet of Glymin^®^ at 7:00 AM this morning. When should he take his third tablet of Glymin^®^?	
	I, N	Number of tablets prescribed	Q12
		How many days of supply were prescribed to Mrs. Lee?	
	I, A	Symptoms of hypoglycemia	Q13
		Which symptom below is related to low blood sugar?	
	I, N, E	Wrong drug taken	Q14
		In comparison with the medication that Mr. Lee got from pharmacy for his first visit, he noticed his medication looked different this time. What is the most appropriate explanation about his new finding?	
**Dietary supplement commercial advertisement**	I, L, E	Indication of dietary supplement	Q15
		What is this product advertised for?	
	I, L, D	Information accuracy assessment	Q16
		Which statement is most appropriate about this commercial advertisement?	
	I, L, E	Side effect of dietary supplement	Q17
		How would you describe the side effects of this product?	

Note: L: literacy; N: numeracy; I: information-seeking; D: decision-making; E: evaluation; A: application.

**Table 2:** Psychometric properties of items in the ChMLM-17 based on the 1410 responses.

Item ID	Item Content to be Assessed	Correctness Rate (%)	Item-Total Correlation	Factor Loading on 1-Factor Solution
Q1	External use	80.8	0.320	0.354
Q2	Fixed-dose combination drug	41.6	0.395	0.348
Q3	Dose	79.7	0.402	0.477
Q4	Side effect	71.6	0.433	0.502
Q5	Active ingredient	75.9	0.418	0.486
Q6	Indication of OTC drug	85.0	0.428	0.672
Q7	Direction to use	85.5	0.460	0.741
Q8	Number of total tablets contained	79.9	0.469	0.683
Q9	Expiration date	73.3	0.499	0.669
Q10	Warnings	63.0	0.528	0.631
Q11	Time to take the next drug	81.7	0.370	0.538
Q12	Number of tablets prescribed	88.3	0.401	0.683
Q13	Symptoms of hypoglycemia	63.8	0.412	0.476
Q14	Wrong drug taken	46.6	0.384	0.383
Q15	Indication for dietary supplement	84.3	0.371	0.543
Q16	Information accuracy assessment	69.9	0.398	0.409
Q17	Side effect of dietary supplement	27.1	0.213	0.190

**Table 3:** Psychometric properties of the ChMLM-17 and ChMLM-13.

Psychometric Properties	ChMLM-17	ChMLM-13
Number of section	4	3
Mean (SD) of total score	11.98 (3.68)	9.18 (2.92)
Median (interquartile range) of total score	13 (10–15)	10 (8–11)
Number of total items	17	13
Reliability		
Internal consistency: α value	0.822	0.787
Number of items contributed to error	1	1
Item contributed to measurement error	Q17	Q17
Construct validity		
Number of factor with factor loading ≥0.3	Mainly 2	Mainly 2
ROC curve for cut-off point		
AUC (95% CI of AUC)	0.723 (0.697–0.750)	0.714 (0.687–0.741)
1st better cut-off point	as 12.5	as 9.5
Sensitivity + specificity	0.606 + 0.729 = 1.335	0.594 + 0.74 = 1.334
2nd better cut-off point	as 13.5	as 10.5
Sensitivity + specificity	0.741 + 0.589 = 1.330	0.743 + 0.566 = 1.309
Final cut-off point	13	10

Note: ROC: receiver operating characteristic; AUC: area under the curve.

**Table 4:** Bivariate analyses of the ChMLM-17 total score across groups with different levels of medication literacy and characteristics.

				Stratification as Two Groups					
	Total			High			Low		
Sample Size (*n*)	1410			805			605		
	Mean	SD	*p*-Value	Mean	SD	*p*-Value	Mean	SD	*p*-Value
Demographic Characteristics									
Education group									
≤Not higher education (senior high and less)	10.55	3.96	<0.001	14.14	1.05	<0.001	8.06	3.46	<0.001
≥Higher education (college or more)	13.26	2.86		14.63	1.13		10.04	2.26	
Age group									
<50 year old	13.21	2.59	<0.001	14.52	1.13	0.068	10.01	2.36	<0.001
≥50 year old	10.40	4.22		14.36	1.11		7.86	3.46	
Area									
North (PeiJiYi and TaoChuMiao)	12.90	2.94	<0.001	14.53	1.09	0.221	9.56	2.72	<0.001
Other areas	11.52	3.92		14.43	1.15		8.36	3.38	
Religious belief									
Not having religious beliefs	12.58	3.25	<0.001	14.46	1.13	0.852	9.08	2.99	0.051
With religious beliefs	11.67	3.85		14.48	1.13		8.51	3.36	
Language									
Not mainly Mandarin	8.69	4.68	<0.001	14.29	1.13	0.367	6.69	3.74	<0.001
Mainly Mandarin	12.28	3.42		14.48	1.13		9.00	3.06	
Student status									
Not student	11.72	3.78	<0.001	14.47	1.12	0.855	8.60	3.29	0.004
Student	14.01	1.72		14.49	1.15		10.75	1.45	
**Self-reported health care utilization**									
Medical care utilization in the past 3 months									
Never use	12.23	3.36	0.092	14.40	1.12	0.229	8.99	3.00	0.105
Ever made doctor visit	11.87	3.81		14.50	1.13		8.53	3.37	
Currently taking medication									
No	12.64	3.11	<0.001	14.48	1.12	0.815	9.34	2.79	<0.001
Yes	10.95	4.25		14.46	1.14		7.94	3.59	
Need help in taking medications									
No	11.77	3.77	0.054	14.45	1.11	0.020	8.54	3.29	0.576
Yes	9.94	4.43		15.75	1.50		8.00	3.13	
Use complementary and alternative medicine									
No	10.09	4.51	<0.001	14.23	1.03	0.035	7.48	3.86	<0.001
Yes	12.35	3.37		14.50	1.14		9.03	2.97	
**Self-reported health literacy**									
Perception of knowing name(s) of the taken medications									
<50%	11.40	4.05	<0.001	14.43	1.09	0.211	8.14	3.52	<0.001
≥50%	12.88	2.78		14.53	1.17		9.81	2.25	
Perception of knowing effect(s) of the taken medications									
<50%	10.85	4.14	<0.001	14.37	1.06	0.076	8.04	3.50	<0.001
≥50%	12.74	3.11		14.52	1.15		9.35	2.85	
Have difficulty understanding information provided by healthcare professionals									
Usually and always	9.91	4.32	<0.001	14.05	0.96	0.016	7.33	3.50	<0.001
Otherwise	12.14	3.58		14.49	1.13		8.83	3.20	
Have difficulty asking medication related problems to healthcare professionals				14.47	1.12				
Usually and always	10.16	4.36	0.001	14.08	1.02	0.071	7.41	3.62	0.015
Otherwise	12.07	3.63		14.48	1.13		8.75	3.23	
Have difficulty taking medications									
Usually and always	10.45	4.69	0.010	14.34	1.23	0.544	7.03	3.85	0.003
Otherwise	12.05	3.61		14.47	1.12		8.76	3.21	
Understand the medication labels of prescription									
<50%	7.34	4.54	<0.001	13.97	0.82	0.014	5.85	3.59	<0.001
≥50%	12.57	3.10		14.49	1.13		9.43	2.71	
Understand the medication package/box labels									
<50%	7.64	4.51	<0.001	14.18	0.92	0.135	6.14	3.56	<0.001
≥50%	12.60	3.08		14.48	1.13		9.46	2.73	
Understand the package inserts									
<50%	8.97	4.43	<0.001	14.25	0.96	0.086	7.07	3.56	<0.001
≥50%	12.66	3.11		14.49	1.14		9.41	2.83	
Fill out medical forms by yourself									
Confident	12.48	3.20	<0.001	14.49	1.13	0.055	9.27	2.78	<0.001
Not confident	8.63	4.82		14.18	1.03		6.47	3.89	
Understand written instructions provided by hospitals									
Confident	12.47	3.23	<0.001	14.50	1.14	0.053	9.24	2.81	<0.001
Not confident	9.77	4.69		14.26	1.01		7.04	3.87	

## Data Availability

The study materials and the detail of all analyses are available from the corresponding author upon reasonable request.

## Supplementary Materials

The following are available online at https://www.mdpi.com/1660-4601/17/19/6951/s1, File S1: The questionnaire of the Chinese Medication Literacy Measurement (ChMLM), File S2: The questionnaire of the Medication Literacy Measure (MLM), File S3: The answer key and scoring of the Medication Literacy Measure, File S4: Sociodemographic characteristics of the participants (*n* = 1410), File S5: Evaluation of the participants’ self-reported health care utilization (*n* = 1410), File S6: Evaluation of the participants’ self-reported health literacy (*n* = 1410).
