# Supplementary material for: Conceptualization, Development and Psychometric Evaluations of a New Medication-Related Health Literacy Instrument: The Chinese Medication Literacy Measurement
Source: Int J Environ Res Public Health. 2020 Sep 23;17(19):6951. doi: 10.3390/ijerph17196951 (PMC7579017; doi:10.3390/ijerph17196951)
Supplement: Supplementary file 1 [file ijerph-17-06951-s001.pdf]

International Journal of Environmental Research and Public Health

MDPI

Supplementary Material:

File S1: The questionnaire of the Chinese Medication Literacy Measurement (ChMLM)

中文用藥素養量表

用藥素養的定義是:「個人於獲取、理解、溝通、計算、處理各種不同來源的用藥相關資訊（例如：書面資料、口頭敘述和視覺影像）的程度，藉由應用這些和其自身需求相關的藥品資訊，進行個別藥物使用決策，以保障其用藥安全」。這個量是根據台灣衛生福利部的研究資助「建置民眾用藥安全知識網」之健康素養子計畫，進行相關研究後修訂和改編而成。

這個評估表包含四個部分，包括：(1) 藥品使用相關字彙、(2) 非處方藥品使用標示、(3) 健康食品廣告識讀；與(4) 處方藥品使用標示。這份量表必須依照原作者的建議順序及所附之圖件以紙本或電腦方式進行作答。標準答案及評分依據請參照英文版本最後所附的說明。若有問題請聯絡研究室信箱 2011cpe@gmail.com。

這份中文用藥健康素養量表(ChMLM)及英文版本(MLM-17)之著作權歸屬於主要著作人林香汶博士，所有使用權必須依照台灣著作權法規定辦理。若需使用請事先與林香汶博士聯絡 2011cpe@gmail.com。

第一部分：字彙

下列有關藥物資訊相關的字彙，你可能會在藥盒、藥袋、仿單/藥品說明書或用藥指導單張上看到，針對每一個字彙後面所附的定義/意思，請您判斷其正確與否，若您真的不知道答案正確與否，請勾選「不知道 」

	正確	不正確	不知道
1. 外用: 不是從嘴巴服用	○	○	○
2. 複方: 重複處方	○	○	○
3. 劑量: 藥物使用的量 (如一次服用500毫克)	○	○	○
4. 副作用: 額外的治療效果	○	○	○
5. 成分: 決定 "藥物" 作用之內容物的組成及其所包含的量或比例	○	○	○

第二部分：非處方藥

填答說明：請依據以下所提供之藥盒及說明書**(**仿單**)**上之用藥資訊，回答下列問題並選出最適當的答案。

張先生為 **35** 歲上班族，今天因為身體不舒服，自行到藥局購買藥品，以下是他所購買的藥品的藥盒資訊：

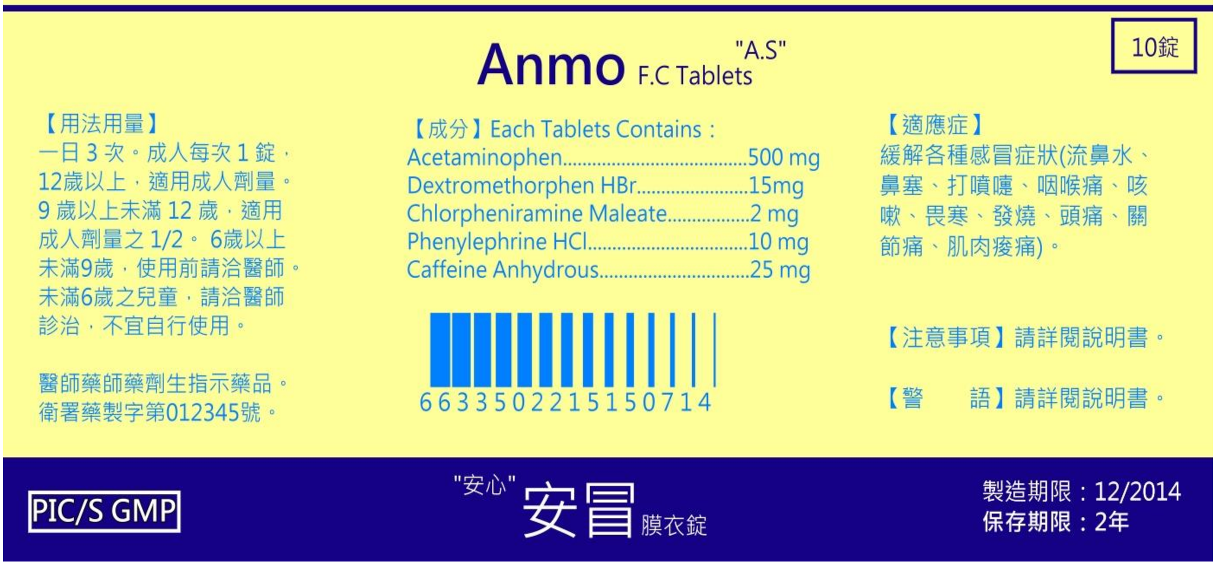

1. 依據上述"藥盒"，張先生可能是因為什麼疾病或症狀而至藥局購買藥物?
   - A. 暈車
   - B. 胃不舒服
   - C. 咳嗽、流鼻水
   - D. 我不知道/我不確定

2. 依藥盒上的"用法用量"說明，請問張先生應該要如何使用這個從藥局購買來的藥物呢?
   - A. 一天 2 次，每次一錠
   - B. 一天 3 次，每次一錠
   - C. 一天 3 次，每次二分之一錠
   - D. 我不知道/我不確定

3. 依"藥盒外觀"看來，這個藥盒應該含有多少粒的藥錠?
   - A. 5 錠
   - B. 10 錠
   - C. 20 錠
   - D. 我不知道/我不確定

4. 依藥盒外觀看來，張先生所買的藥物在正常保存情況下可以放到什麼時候呢?
   - A. 2014 年 12 月底之前
   - B. 2015 年 12 月底之前

Copyright © 2020 reproduced with the copyright owner’s permission

C. 2016 年 12 月底之前

D. 我不知道/我不確定

Copyright © 2020 reproduced with the copyright owner's permission

張先生為 **35** 歲上班族，今天因為身體不舒服，自行到藥局購買藥品，以下是他所購買的藥品的仿單 **(**說明書**)**：

【警　　語】

1. 服用後，若有發疹、發紅、噁心、嘔吐、食慾不振、頭暈、耳鳴、喉嚨疼痛、心跳加速、排尿困難、視覺模糊等症狀時，應停藥立即就醫。
2. 除非有醫師藥師藥劑生指示，孕婦及授乳婦不建議自行使用、三歲以下不建議自行使用、曾經因藥物引起過敏症狀者不得使用。
3. 不要服用超過建議劑量，因高劑量會產生焦慮、暈眩、失眠或肝損傷，兒童可能會產生躁動。
4. 不要服用本藥超過七日。
5. 本藥含 Acetaminophen，應注意下列事項：
   - ✧ 每一顆 Acetaminophen 含量是 500 毫克，每 24 小時內不可超過 4000 毫克。
   - ✧ 若每日喝三杯或更多之酒精性飲料，請詢問醫師是否能服用含有 Acetaminophen 的本藥，因為 Acetaminophen 可能造成肝損害。建議不得與酒精併用，
6. 本藥含 Dextromethorphan，應注意下列事項
   - ✧ 服用 Monoamine oxidase inhibitor (MAOI)或血清素回收抑制劑型的抗憂鬱劑[Selective serotonin reuptake inhibitors (SSRI)]者，不得使用本藥。
7. 本藥含 Chlorpheniramine，應注意下列事項
   - ✧ 服用本藥後，可能引起嗜睡現象，駕車或操作危險器械時要小心。不要飲用含酒精之飲料，亦不要服用安眠藥、鎮定劑之類的藥品。
8. 本藥含 Phenylephrine Hydrochloride， 應注意下列事項：
   - ✧ 除非有醫師藥師藥劑生指示，有心臟病、高血壓、甲狀腺疾病、糖尿病、前列腺腫大引起之排尿困難者不得服用本藥。
   - ✧ 服用 Monoamine oxidase inhibitor (MAOI)期間，或停用 MAOI 兩星期內，不得服用本藥， 如不了解服用之藥物是否含有 MAOI，請詢問醫師或藥師。
9. 本藥含咖啡因類成分，應限制再服用咖啡因藥品、飲料(如：茶、咖啡、可樂等)，過多的咖啡因會引起神經緊張、興奮與失眠，且常會引起心搏過速。

【儲藏條件】30℃ 以下，密封儲存

【包　　裝】10 錠 PVC 鋁箔盒裝。

安心製藥股份有限公司

台北市安心區安心路一段 **1** 號

Copyright © 2020 reproduced with the copyright owner’s permission

5. 依仿單(藥品說明書)之"警語"來看，張先生服用所買的藥物後，應該要注意的情況，下列敘述何者"最為正確"?
   A. 接受任何手術或拔牙前,請告知醫師或牙醫師正在服用本藥
   B. 每日(24 小時內)不可使用超過 4000 毫克(8 顆)
   C. 服用本藥時應避免與葡萄柚或葡萄柚汁併服
   D. 我不知道/我不確定

Copyright © 2020 reproduced with the copyright owner's permission

第三部分：廣告說詞

填答說明：請依據以下所敘述的用藥相關資訊，回答下列問題並選出最適當的答案。

以下產品廣告內容**…**『你有過那種「渾身骨頭都快拆了」的經驗嗎？**……**服用◯◯產品六個月後，你就不用 再穿鐵衣或護膝、也不需要使用其他會傷胃的產品**(**如止痛藥**)**、更可以丟掉昂貴的維骨力，因為這個產品　 會讓你的關節不痛、骨頭壯壯喔！**…**』

1. 這個產品 "最主要" 是用來做甚麼用的？
   A. 緩解腸胃不舒服
   B. 緩解骨關節不舒服
   C. 解肌肉痠痛不舒服
   D. 我不知道/我不確定

2. 針對這個廣告內容，您對此產品的看法是？
   A. 這個產品比處方藥及其他健康食品還有效
   B. 這個產品有臨床研究證實其臨床效果(作用)
   C. 這個廣告內容可能是錯誤的
   D. 我不知道/我不確定

3. 針對這個廣告內容，下列何者 "最適合" 描述或隱射有關於此產品的『副作用』？
   A. 2014 年 12 月底之前
   B. 2015 年 12 月底之前
   C. 2016 年 12 月底之前
   D. 我不知道/我不確定

Copyright © 2020 reproduced with the copyright owner's permission

第四部分：處方藥

填答說明：請依據以下所提供之藥袋及藥盒上之用藥資訊，回答下列問題並選出最適當的答案。

蔡先生因為被醫師診斷其患有糖尿病，且決定開始使用藥品治療，醫生開給了他一些藥物，其中一個藥物的藥袋內容資料如下：

XXXXX 醫院
XX 縣/市 OO 區 OO 街 OO 號
電話: (0x) 1234567

調劑日期	104/08/06	領藥號	20034
姓名	蔡 XX	病歷號碼	25688652
生日	050/03/20	性別	男
用法用量	口服<br>每天三次，餐後或隨餐服用，每次 1 錠		
藥名	糖美錠 **500** 毫克	調劑天數	28 天
主要成分	METFORMIN	發藥量	84 錠
外觀標記	錠劑(鋁箔/膠箔)		
適應症	糖尿病		
可能副作用	腸胃不適、腹痛、腹脹、腹瀉、乳酸中毒(虛弱、呼吸困難)		
注意事項/警語	若有低血糖症狀(冒冷汗、心悸、頭痛、痙攣、昏亂等)，應立即吃含糖食物		

科別：新陳代謝科 01 診　處方醫師: 林 XX　調劑藥師: 張 XX

請核對藥袋『姓名』，當面點清『藥品數量及種類』。
其他注意事項請詳閱藥袋背面說明。如有疑問請洽詢藥師。

1. 蔡先生早上服用完了第一錠的「糖美錠」後，第三錠的服用的時間應該是何時呢？
   A. 今天中午
   B. 今天晚上
   C. 明天早上
   D. 我不知道/我不確定

2. 醫師總共幫蔡先生開了幾天的藥量呢？
   A. 3 天
   B. 28 天
   C. 84 天
   D. 我不知道/我不確定

3. 服用此藥物可能會引起和副作用為"低血糖"有關的症狀，例如以下哪一個症狀？

Copyright © 2020 reproduced with the copyright owner’s permission

A. 腹瀉
B. 腹痛
C. 心悸
D. 我不知道/我不確定

Copyright © 2020 reproduced with the copyright owner's permission

第一次領藥從藥局拿回來的藥盒

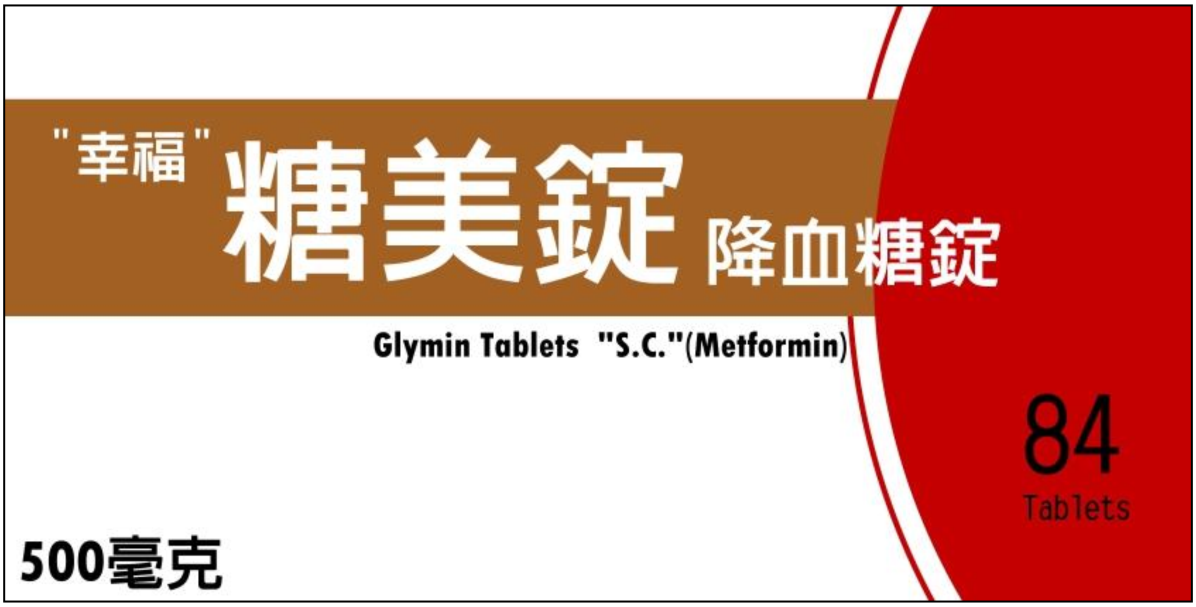

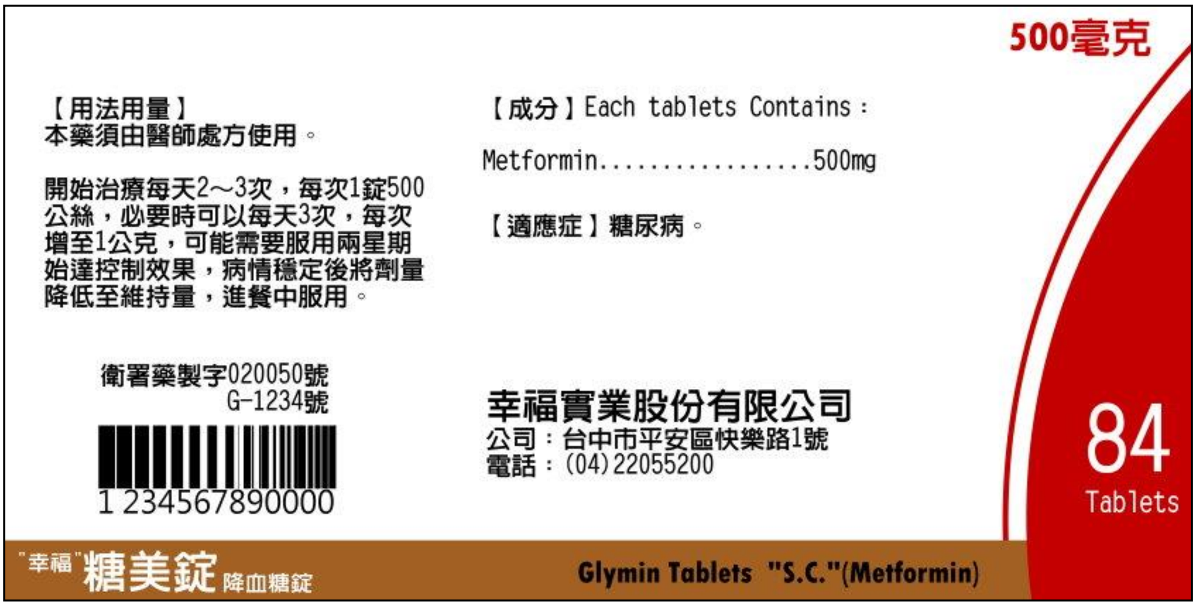

Copyright © 2020 reproduced with the copyright owner's permission

第二次領藥從藥局拿回來的藥盒

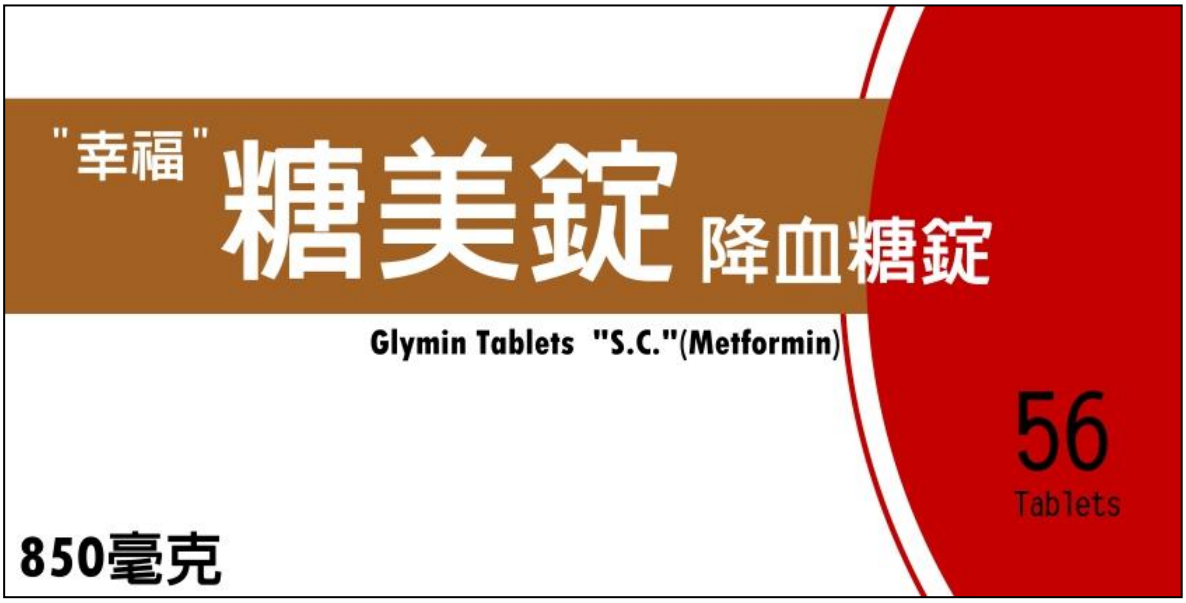

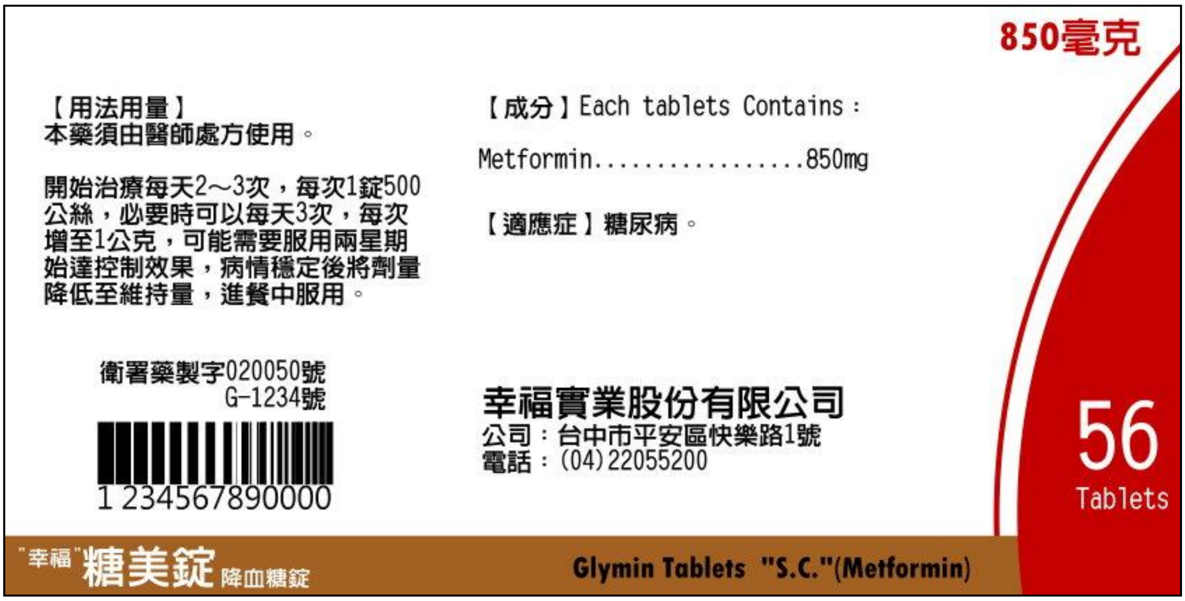

4. 一個月後再去看醫師，醫師告訴蔡先生說因為病情穩定,先維持原來的處方一陣子，但從藥局拿回來的藥盒與之前的比對，蔡先生發現一些現象，下列哪一個選項是你認為"最正確且合理"的反應呢？
   - A. 蔡先生新拿到的藥物每顆的量比較高
   - B. 醫師幫蔡先生改劑量但沒有告知
   - C. 蔡先生的藥品與原來的藥是完全一樣的
   - D. 我不知道/我不確定

Copyright © 2020 reproduced with the copyright owner's permission

File S2: The questionnaire of the Medication Literacy Measure (MLM)

Medication Literacy Measure (MLM-17)

The Medication Literacy Measure (MLM-17) was developed based on the following definition: "Medication literacy is the degree to which individuals can obtain, comprehend, communicate, calculate and process patient-specific information about their medications to make informed medication and health decisions in order to safely and effectively use their medications, regardless of the mode by which the content is delivered (e.g. written, oral and visual)." (Pouliot A, et al., 2018). This measure was revised and adapted based upon the validated 17-item Chinse Medication Literacy Measured (ChMLM-17), sponsored by a research grant from the Ministry of Health and Welfare, Taiwan (Patient Medication Safety Knowledge Network Subproject-Medication Literacy).

The MLM-17 includes four sections: vocabulary of medications, over-the-counter medication labels, dietary supplement commercial advertisement, and prescription labels. The sections should be administered in the fixed order as designed. This MLM can be prepared in the paper-and-pencil format or electronic format, but the corresponding figure(s) should be attached respectively.

The copyright of the Chinese Medication Health Literacy Measure (ChMLM) and MLM-17 (English version of ChMLM) belongs to the principle investigator, Dr. Hsiang-Wen (Margaret) Lin, as the copyright owner. All use rights must be followed in accordance with Copyright Laws. Permission for use is required to contact Dr. Hsiang-Wen (Margaret) Lin in advance at 2011cpe@gmail.com.

Copyright © 2020 reproduced with the copyright owner's permission

Section 1: Vocabulary of medications

Note: Please indicate "Don't know", if you do not know the answer

The following vocabularies are commonly used on medication bottles, packaging, package inserts, and patient education brochures. Please try your best to differentiate whether the explanation next to each vocabulary is "true" or "false". If you do not know the answer, please select "don't know".

		True	False	Don't know
1.	External use: should not be taken by mouth	○	○	○
2.	Fixed-dose combination drug: duplicate prescriptions	○	○	○
3.	Dose: the quantity of medication that is taken at one time (e.g. take 500mg at a time)	○	○	○
4.	Side-effect: extra benefits of the treatment	○	○	○
5.	Active ingredient: the main component of the medication that provides a therapeutic effect	○	○	○

Copyright © 2020 reproduced with the copyright owner's permission

Section 2: Over-the-counter medication labels

<u>Note: Indicate "I do not know" if you do not know the answer</u>

Hypothetical scenario:
Mr. Chang, a 40-year-old office staff working in a company, is using an over-the counter medication to relieve his discomfort. Please answer the following 5 questions based on the attached information on the medication package label and package insert label listed below.

The package label of this over-the-counter medication:

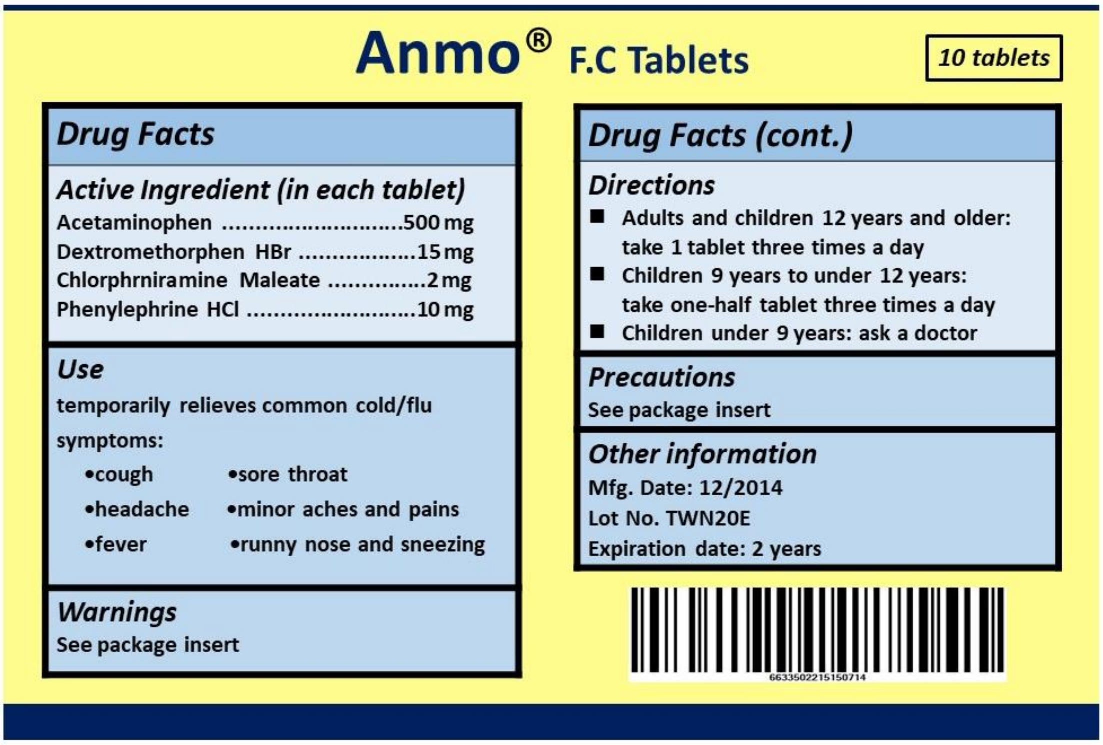

1. What is this medication used for?
    A. Motion sickness
    B. Stomach upset
    C. Cough and runny nose
    D. I do not know
2. How should Mr. Chang take this medication?
    A. 1 tablet twice a day
    B. 1 tablet three times a day
    C. Half a tablet three times a day
    D. I do not know
3. How many tablets are in this package according to the package label?
    A. 5 tablets
    B. 10 tablets
    C. 20 tablets
    D. I do not know
4. What is the expiration date of this medication?
    A. December 2014
    B. December 2015
    C. December 2016
    D. I do not know

Copyright © 2020 reproduced with the copyright owner's permission

The package insert label of this over-the-counter medication is shown below:

【 **Warnings**】

1. Do not use if you are allergic to acetaminophen, dextromethorphan, chlorpheniramine, phenylephrine, or caffeine.
2. Ask a doctor or pharmacist before use if you are pregnant or breast-feeding.
3. When using this product do not exceed recommended dosage.
4. Stop use and ask a doctor if you get a rash, redness, nausea, vomiting, stomach upset, dizzy, drumming in the ears, throat pain, palpitation, difficult urinating, or blurred vision
5. Stop use and ask a doctor if your symptoms get worse or last more than 7 days.
6. This product contains acetaminophen. Severe liver damage may occur if you take
   (1) more than 8 tablets in 24 hours, which is the maximum daily amount
   (2) 3 or more alcoholic drinks daily while using this product
7. This product contains dextromethorphan and phenylephrine, and you should use it with caution if you are taking a prescription monoamine oxidase inhibitor (MAOI) (a type of drug used for depression or Parkinson's disease), or for 2 weeks after stopping an MAOI drug.
8. This product contains chlorpheniramine, and marked drowsiness may occur. When using this product, avoid alcoholic beverages and be careful when driving a motor vehicle or operating machinery.

【**Other information**】store at room temperature 20-25°C (68-77°F)

5. Based on the warnings on the package insert label, which situation listed below should Mr. Chang pay more attention to when using this medication?
   A. Inform the doctor(s) about his use of this medication before doing surgery
   B. Do not take more than 8 tablets in 24 hours
   C. Avoid taking this medication with grape fruit juice
   D. I do not know

Copyright © 2020 reproduced with the copyright owner's permission

Section 3: Dietary supplement commercial advertisement

Note: Please indicate "I do not know", if you are unsure of the answer.

Please answer the following two questions based on the commercial advertisement statement below:

"Have you ever had a feeling that all your bones fell apart? ……After taking BoneJoinBetter® product for six months, you will no longer need to wear braces or take pain medications harmful to your stomach. Moreover, you can even throw away other expensive dietary supplements away. That is because BoneJoinBetter® can get rid of your knee pain and strengthen your bones even better!"

Please answer the following two questions based on this commercial advertisement statement.

1. What is this product advertised for?
   - A. To relieve an upset stomach
   - B. To relieve pain of bone and joint
   - C. To relieve stiffness of muscle
   - D. I do not know

2. Which statement is most appropriate about this commercial advertisement?
   - A. This product is more effective than prescription drugs or other dietary supplements
   - B. There is clinical evidence to support the benefit of this product
   - C. This advertisement may contain false information
   - D. I do not know

3. How would you describe the side effects of this product?
   - A. This product does no harm to the stomach
   - B. This product has no side effects
   - C. Compared with other expensive products, this product has fewer side effects
   - D. I do not know

Copyright © 2020 reproduced with the copyright owner's permission

Section 4: Prescription labels

David. C. Lee visited a doctor for his diabetes control. Below is one of his prescription labels.

Please answer the following three questions accordingly.

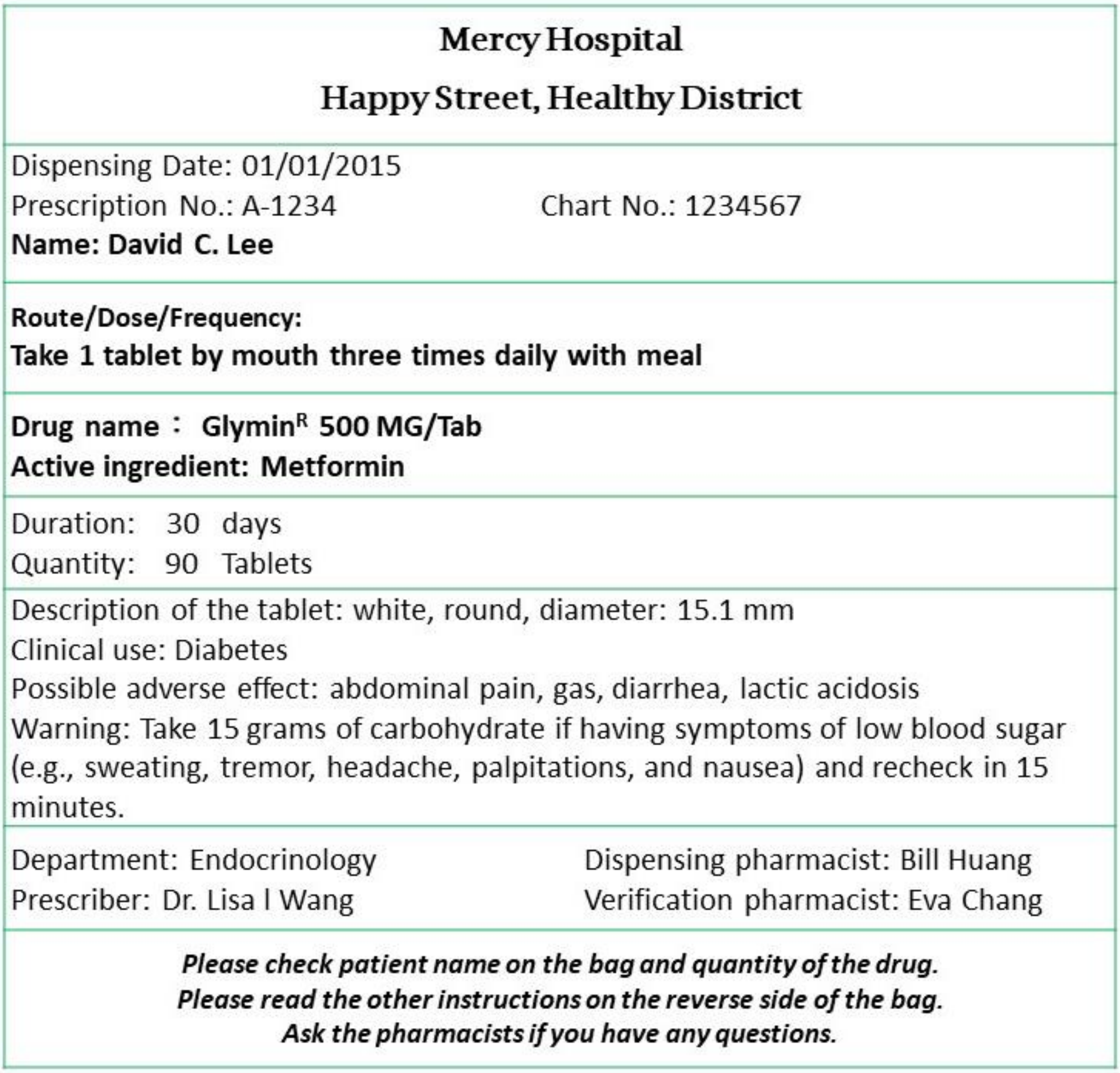

Mercy Hospital

Happy Street, Healthy District

Dispensing Date: 01/01/2015
Prescription No.: A-1234 Chart No.: 1234567
Name: David C. Lee

Route/Dose/Frequency:
Take 1 tablet by mouth three times daily with meal

Drug name： Glymin^R 500 MG/Tab
Active ingredient: Metformin

Duration: 30 days
Quantity: 90 Tablets

Description of the tablet: white, round, diameter: 15.1 mm
Clinical use: Diabetes
Possible adverse effect: abdominal pain, gas, diarrhea, lactic acidosis
Warning: Take 15 grams of carbohydrate if having symptoms of low blood sugar (e.g., sweating, tremor, headache, palpitations, and nausea) and recheck in 15 minutes.

Department: Endocrinology Dispensing pharmacist: Bill Huang
Prescriber: Dr. Lisa I Wang Verification pharmacist: Eva Chang

Please check patient name on the bag and quantity of the drug.
Please read the other instructions on the reverse side of the bag.
Ask the pharmacists if you have any questions.

1. Mr. Lee took his first tablet of Glymin® at 7:00 AM this morning. When should he take his third tablet of Glymin®?
   - A. With or after lunch at noon on the same day
   - B. With or after dinner in the evening of the same day
   - C. With or after breakfast tomorrow morning
   - D. I do not know

2. How many days of supply were prescribed to Mr. Lee?
   - A. 3 days
   - B. 30 days
   - C. 90 days
   - D. I do not know

Copyright © 2020 reproduced with the copyright owner's permission

3. Which symptom below is related to low blood sugar?
   - A. Abdominal pain
   - B. Diarrhea
   - C. Palpitations
   - D. I do not know

Below is the medication package that Mr. Lee received from the pharmacy after <u>his first doctor's visit</u>.

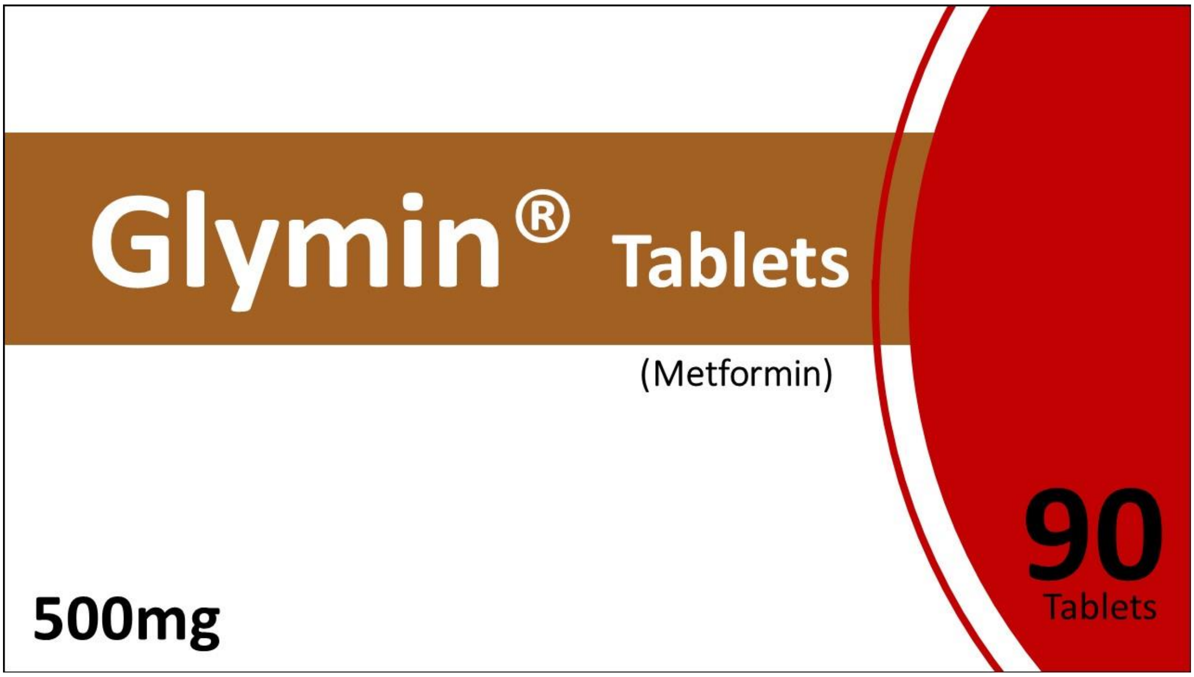

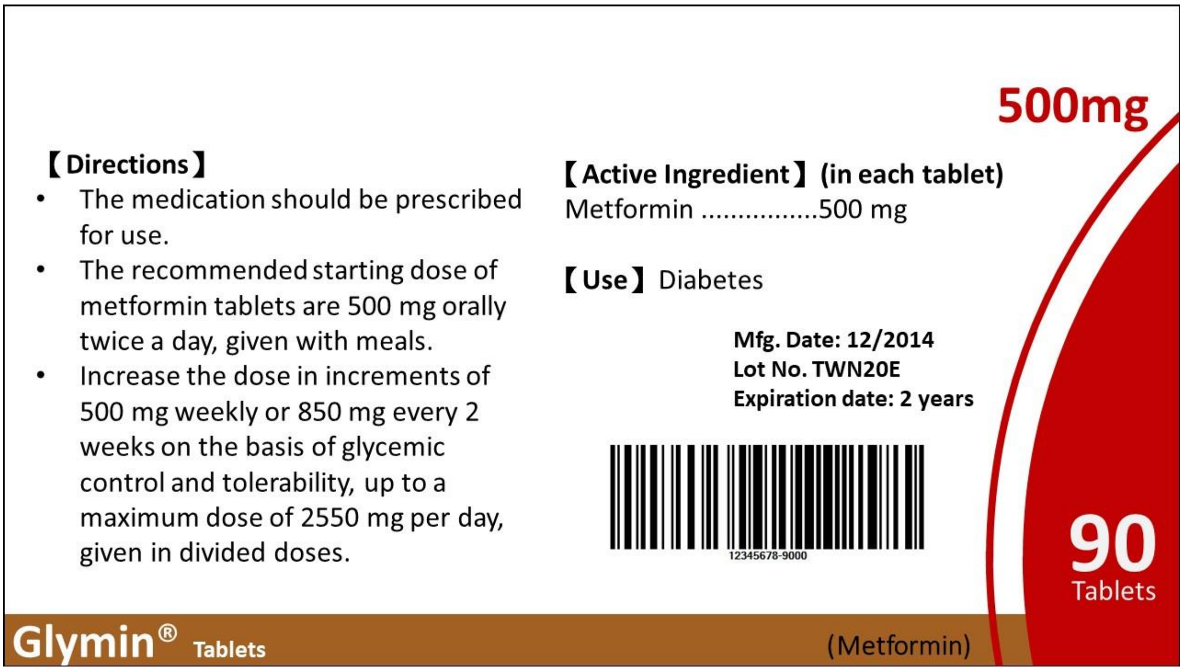

Copyright © 2020 reproduced with the copyright owner's permission

One month later, Mr. Lee visited the doctor again. The doctor told Mr. Lee that his blood sugar was under good control and reminded him to take the same dose of medication.
Below is the medication that Mr. Lee got from the pharmacy for <u>his follow-up visit</u>.

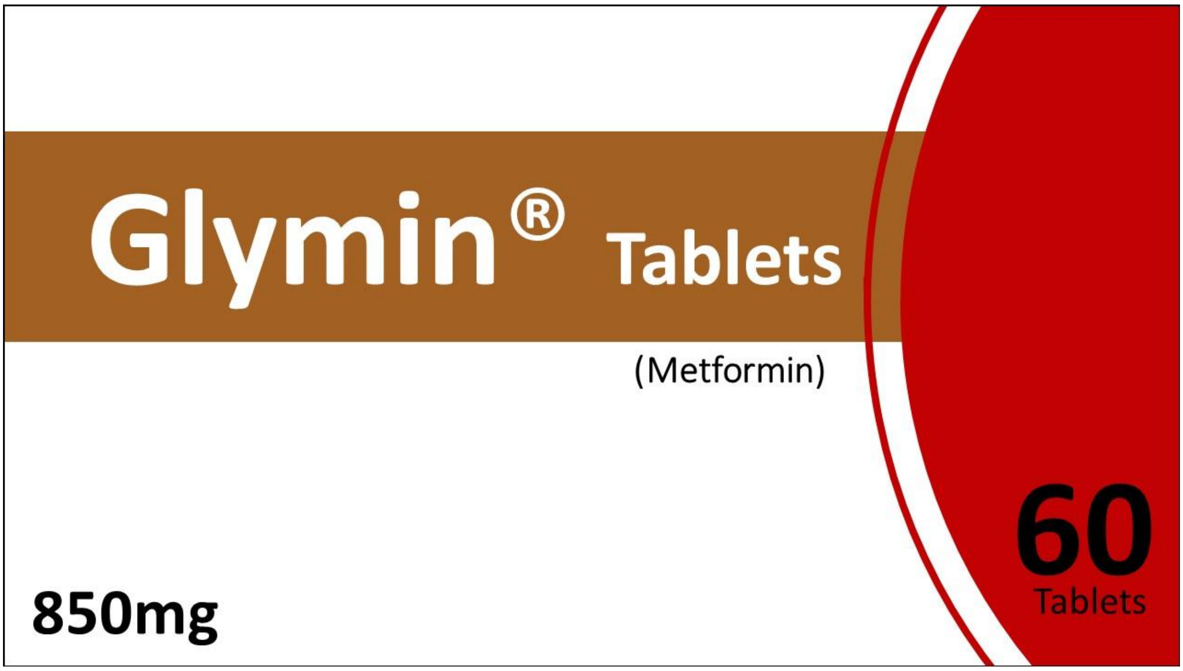

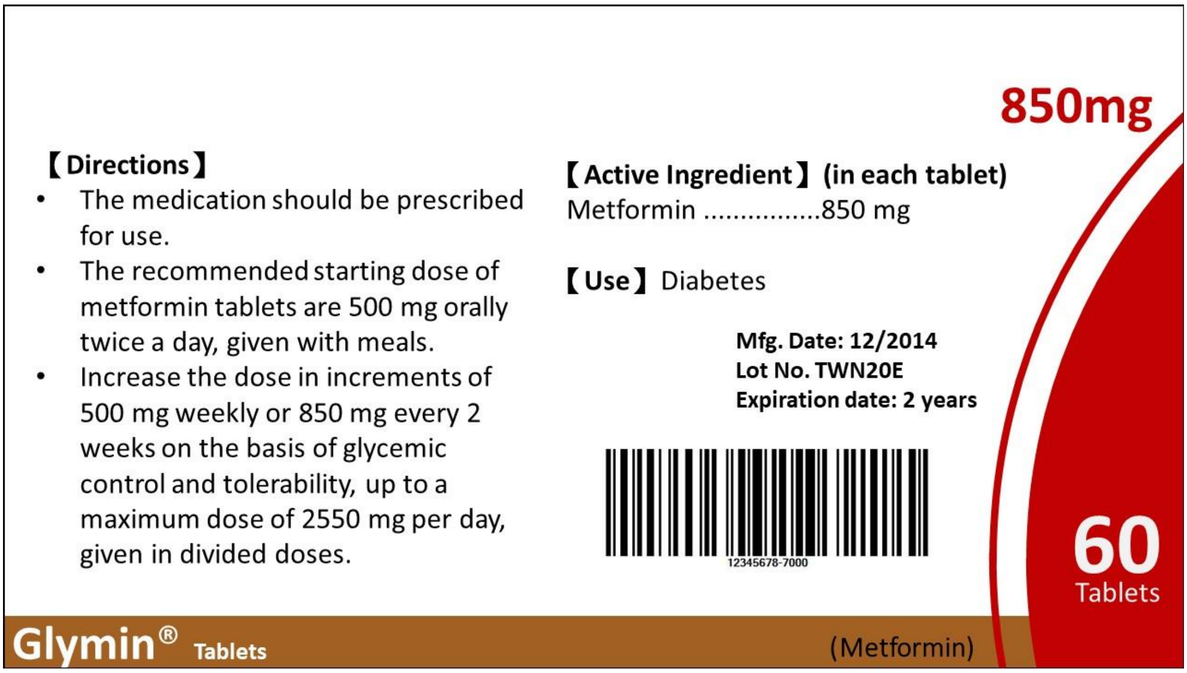

4 In comparison with the medication that Mr. Lee got from pharmacy for his first visit, he noticed his medication looked different this time. What is the most appropriate explanation about his new finding?

A. The new medications have a higher strength than the previous medications.
B. The doctor decided to change his prescription dose of medication without any notice.
C. The new medications have the same strength as the previous medications
D. I do not know

File S3: The answer key and scoring of the Medication Literacy Measure

Instruction of scoring for Medication Literacy Measure (MLM)

This MLM is recommended to be used by healthcare professionals. The correct answers, its scoring and cut-off point to differentiate high or low medication literacy level should be separated from the MLM-17 when administered. The section 4: prescription labels can be separately for scoring as MLM-13 score, if section 4 is not applicable. If there is any question, please contact office email at 2011cpe@gmail.com.

Answers:

Section 1 (Vocabulary of medications with 5 items): True, False, True, False, True

Section 2 (Over-the-counter medication labels with 4 items): C, B, B, C, B

Section 3 (Dietary supplement commercial advertisement with 3 items): B, C, A

Section 4 (Prescription labels with 4 items): B, B, C, A

Scoring:

Correct answer is given 1 point;

Incorrect answer is given 0 point;

MLM-17 score is the combined score of all four sections of the MLM. Please add up points from all four sections of the MLM to get the total MLM-17 score. The range of possible total MLM-17 score is 0 to 17 point. A total MLM-17 score of 13 or higher indicates "**high medication literacy**". A total MLM-17 score of 12 or less indicates "**low medication literacy**".

MLM-13 score is the combined score of Section 1 through Section 3 (Section 4 is excluded from scoring). Please add up points from the first three sections of the MLM-17 to get the total MLM-13 score. The range of possible total MLM-13 is 0 to 13. A total MLM-13 score of 10 or higher indicates "**high medication literacy**". A total MLM-13 score of 9 or less indicates "**low medication literacy**".

The obtained scores can be treated as a continuous measure or dichotomized as < 13 or ≥ 13 for the MLM-17 or < 10 or ≥ 10 for the MLM-13, respectively.

The copyright of the Chinese Medication Health Literacy Measure (ChMLM) and MLM-17 (English version of ChMLM) belongs to the principle investigator, Dr. Hsiang-Wen (Margaret) Lin, as the copyright owner. All use rights must be followed in accordance with Copyright Laws. Permission for use is required to contact Dr. Hsiang-Wen (Margaret) Lin in advance at 2011cpe@gmail.com.

File S4. Sociodemographic characteristics of the participants (*n* = 1410).

Variables	n (%) or Mean (S.D.)
**Age**	45.18 (17.05)
**Gender**	
Female	827 (58.65)
Male	577 (40.92)
Missing	6 (0.43)
**Education**	
Elementary school or less	139 (9.86)
Junior high school	123 (8.72)
Senior high school	403 (28.58)
Bachelor's degree or a 4-year college degree	582 (41.28)
Graduate degree or above	141 (10.00)
Others	16 (1.13)
**Living area**	
Northern Taiwan	471 (33.40)
Middle Taiwan	432 (30.64)
Southern Taiwan	467 (33.12)
Eastern Taiwan	36 (2.55)
Other island	1 (0.07)
**Religion**	
Taoism	521 (36.95)
Buddhism	180 (12.77)
Christianity	83 (5.89)
Esoteric Buddhism	77 (5.46)
I-Kuao Tao	24 (1.70)
Catholicism	22 (1.56)
Islam	4 (0.28)
Non-religion	485 (34.40)
**Language**	
Mainly Mandarin	1292 (91.63)
Not mainly Mandarin	118 (8.37)
**Occupation**	
Service	310 (21.99)
Housekeeper	184 (13.05)
Student	158 (11.21)
Military, government, or education	144 (10.21)
Manufacturing	131 (9.29)
Freelance	107 (7.59)
Health care	58 (4.11)
Finance	54 (3.83)
Information technology	28 (1.99)
Others	230 (16.31)
**Annual individual income**	
Less than US$ 5,000	639 (45.32)
Between US$ 5,001-10,000	226 (16.03)
Between US$ 10,000-15,000	188 (13.33)
Between US$ 15,001-20,000	170 (12.06)
More than US$ 20,000	181 (12.84)
Missing	6 (0.43)

File S5: Evaluation of the participants' self-reported health care utilization (*n* = 1410)

Items	n (%)
**Frequency of medical care utilization over the past 3 months**	
Never	435 (30.85)
Once	537 (38.09)
Once a month	320 (22.70)
Once a week	82 (5.82)
Several times a week	26 (1.84)
Missing	10 (0.71)
**Currently taking medication**	
No	860 (60.99)
Yes	544 (38.58)
Missing	6 (0.43)
**Need help in taking medications**	
No	1219 (86.45)
Yes	16 (1.13)
Missing	175 (12.41)
**Use of complementary or alternative medications**	
Over-the-counter medication	616 (43.69)
Other alternative medication	561 (39.79)
None	233 (16.52)

File S6: Evaluation of the participants' self-reported health literacy (*n* = 1410)

Items	n (%)
**Know the name(s) of taken medications**	
Fully	141 (10.00)
Almost	227 (16.10)
Half	181 (12.84)
Somewhat	346 (24.54)
Not at all	462 (32.77)
Missing	53 (3.76)
**Understand the effect(s) of taken medications**	
Fully	234 (16.60)
Almost	361 (25.60)
Half	248 (17.59)
Somewhat	311 (22.06)
Not at all	199 (14.11)
Missing	57 (4.04)
**Have difficulty understanding information provided by healthcare professionals**	
Always	25 (1.77)
Often	78 (5.53)
Sometimes	342 (24.26)
Rarely	507 (35.96)
Never	457 (32.41)
Missing	1 (0.07)
**Have difficulty asking medication related questions to healthcare professionals**	
Always	13 (0.92)
Often	49 (3.48)
Sometimes	213 (15.11)
Rarely	407 (28.87)
Never	727 (51.56)
Missing	1 (0.07)
**Have difficulty taking medications**	
Always	11 (0.78)
Often	47 (3.33)
Sometimes	218 (15.46)
Rarely	495 (35.11)
Never	635 (45.04)
Missing	4 (0.28)
**Understand the information labeling on prescription medications**	
Fully	414 (29.36)
Almost	635 (45.04)
Half	203 (14.40)
Somewhat	109 (7.73)
Not at all	49 (3.48)
**Understand the information on medication package label**	
Fully	339 (24.04)
Almost	661 (46.88)
Half	233 (16.52)
Somewhat	116 (8.23)
Not at all	60 (4.26)
Missing	1 (0.07)
**Understand the information on package insert**	
Fully	269 (19.08)

Almost	570 (40.43)
Half	310 (21.99)
Somewhat	169 (11.99)
Not at all	88 (6.24)
Missing	4 (0.28)
**Fill out medical forms by yourself**	
Very confident	297 (21.06)
Somewhat confident	931 (66.03)
A little confident	145 (10.28)
Not confident at all	37 (2.62)
**Understand the printed information provided by clinic/hospital**	
Very confident	227 (16.10)
Somewhat confident	928 (65.82)
A little confident	206 (14.61)
Not confident at all	48 (3.40)
Missing	1 (0.07)
